# Radiation Exposure of Patient and Operating Room Personnel by Fluoroscopy and Navigation during Spinal Surgery

**DOI:** 10.1038/s41598-019-53472-z

**Published:** 2019-11-27

**Authors:** G. Bratschitsch, L. Leitner, G. Stücklschweiger, H. Guss, P. Sadoghi, P. Puchwein, A. Leithner, R. Radl

**Affiliations:** 1 Department of Orthopedics and Trauma, Medical University of Graz, Graz, Austria; 2 Competence Center for Medical Physics and Radiation Protection, Medical University of Graz, Graz, Austria

**Keywords:** Orthopaedics, Risk factors

## Abstract

Intraoperative radiography imaging is essential for accurate spinal implant placement. Hazards caused by ionizing radiation raised concern on personnel’s work life long exposure in the operating room (OR). To particularize a cumulative risk estimation of radiation of personnel and patient, depending on used methods (C-arm fluoroscopy, O-arm navigation) and patient characteristics during spinal surgery, detailed investigation of radiation exposure in a clinical setting is required. Lumbosacral dorsal spinal fusion was performed in 37 patients (19 navigated, 18 fluoroscopy) during this prospective study. Radiation exposure was measured on several body regions with thermoluminescent dosimeters on patient and OR personnel (surgeon, assistant, sterile nurse, radiology technologist). Comparison between patient characteristics and radiation exposure was included. The highest patients values were measured in the surgery field and gonads area during navigation (43.2 ± 19.4 mSv; fluoroscopy: 27.7 ± 31.3 mSv; p = 0.02), followed by the thoracic region during fluoroscopy (7.7 ± 14.8 mSv; navigation: 1.1 ± 1.0 mSv; p = 0.06), other measured regions can be considered marginal in comparison. Amongst OR personnel exposure of the surgeon was significant higher during fluoroscopy (right hand: 566 ± 560 µSv and thoracic region: 275 ± 147 µSv; followed by thyroid and forehead) compared to navigation (right finger: 49 ± 19 µSv; similar levels for all regions; p < 0.001 in all regions). When compared to the surgeon, other OR personnel had significantly lower radiation doses on all body regions using fluoroscopy, and similar dose during navigation. The highest eye’s lens region value was measured during fluoroscopy for the patient (185 ± 165 µSv; navigation: 205 ± 60 µSv; p = 0.57) and the surgeon (164 ± 74 µSv; navigation: 92 ± 41 µSv; p < 0.001). There was a significant correlation between patient BMI and radiation exposure to the surgery field during fluoroscopy. To our knowledge, these data present the first real life, detailed comparison of radiation exposure on OR personnel and patients between clinical use of navigation and fluoroscopy. Although patient’s radiation dose is approximately 3-fold during navigation compared to the fluoroscopy, we found that a spinal surgeon could perform up to 10-fold number of surgeries (10.000 versus 883) until maximum permissible annual effective radiation dose would be reached. Especially for a spinal surgeon, who is mainly exposed amongst OR personnel, radiation prevention and protection must remain a main issue.

## Introduction

Recent reports on 13% increase of cancer risk amongst members of the Scoliosis Research Society^1^ aroused worries amongst medical workers in the field of spinal surgery, concerning their own and their patients’ health risk caused by radiographic imaging. Furthermore, in a small Italian hospital, where radiation protection practice was poor, a retrospective study revealed a cancer incidence of 29% (9 in 31) in orthopaedic surgeons exposed to medical radiation compared to 4% (7 in 158) in unexposed orthopaedic surgeons^2^. This issue seems even more alarming in the context of a 600% increase of medical radiation that has been reported for the US population since the 1980s, mainly resulting from diagnostic procedures^3^. Less life-threatening, but also health relevant for long time exposure to radiation amongst medical workers, is the dose-dependent induction of cataract in human eyes’ lens tissue^4,5^. This issue seems especially relevant for spine surgeons: Compared to other procedures involving fluoroscopy, radiation exposure of the surgeon is up to 12 times greater during spine surgery^6^.

Findings on these considerable health risks led to a steady reduction of the annual occupational threshold recommendation by the International Commission on Radiological Protection (ICRP) to an effective dose of 20 mSv averaged over 5 years with no single year having more than 50 mSv exposure^7^. According to legal regulations, the limit for dose equivalent to the skin and extremities is 500 mSv per year. The most restrictive limit is to the lens of the eye, where an annual limit of 50 mSv, or 100 mSv within 5 years, has been introduced into the International Atomic Energy Agency (IAEA) Basic Safety Standards (BSS).

Modern innovations may provide an opportunity for the reduction of radiation exposure to the personnel. The use of navigation technology can allow the operating room (OR) staff to leave the OR during exposure, allowing for radiation free implant placement^8^.

Even though several studies with a certain focus on radiation exposure during spinal surgery have been conducted before^9–14^, detailed data of OR personnel and patient exposure in the clinical setting with an additional focus on the comparison of the two most broadly used techniques, fluoroscopy (C-arm) and navigation (O-arm), to our knowledge, has not been published before. We believe that this information is essential for the surgeon to choose the most feasible imaging for planned surgeries with a special focus on reduction of radiation dose for patient and OR staff.

The objective of this study was to (1) closely evaluate usage of fluoroscopy and navigation technology in the clinical setting, concerning radiation exposure of patient and OR personnel, involved in the imaging process during spinal surgery. (2) Further focussing on several body regions should be performed, to allow a statement concerning the relative risk of reaching the maximum working life radiation dose for these imaging methods.

## Methods

### Ethics statement

This study was approved by the Ethics Committee at Medical University of Graz (Reference number: 27–444 ex 14/15 – Amendment (1). All experiments were performed in accordance with relevant guidelines and regulations; informed consent was obtained from all participants.

### Study population

Patients were prospectively enclosed in this study, and randomly assigned to fluoroscopy (n = 18; C-arm, Siemens, Berlin, Germany) or navigation group (n = 19; O-arm, Medtronic, Minneapolis, MN) pursuant to which method was indicated preoperatively by a single senior spine surgeon (RR). All cases underwent a posterior spinal surgical approach for pedicle instrumentation at our institution between 11/2016 and 11/2017 by the same surgeon (RR). During the navigation cases an initial scan was performed prior to navigation and after pedicle screw placement, during which the whole OR personnel left the OR into a radiation shielded area. In non-navigated patients the whole surgery was performed fluoroscopy guided. In both groups, if necessary, interbody device implantation through a posterolateral transforaminal lumbar interbody (TLIF) approach and alignment correction was performed fluoroscopy guided after screw placement, which was not part of the study measurements any more. Anesthesia team left the OR into a shielded area during radiation emission in both study settings and was therefore not included. Patients’ demographic data (age, BMI) and surgery specific data were collected for the study.

### Quantification of radiation

Radiation emission from C-arm and O-arm were calculated in Gray (Gy) and expressed as dose area product (expressed as mGycm^2^). *In-vivo* measurements with lithium fluoride thermoluminescent dosimeters (TLD) were performed. Dosimeters were read out using a Harshaw 6600 Plus automatic TLD reader (Thermo Fisher Scientific, Waltham, MA). Reader calibration was performed by TLDs, which are pre-irradiated from the Austrian Meteorological Office (Hp (0.07)) and for all TLDs a separate element correction coefficient was used to determine the skin radiation dose. In respect of this procedure, due to the directionality of dosimeters and energy spectrum of the scattered radiation, the overall uncertainty of the measurements is 27.5% (k = 1). The *in-vivo* measurements were performed in specific locations (forehead, eye lens area/glasses, thyroid region, caudal thoracic region, right/left 2^nd^ finger (surgeon only), abdominal/gonads area (patient only)) outside of any lead aprons and thyroid collar worn by the OR staff (surgeon, assisting surgeon, sterile nurse, radiology technologist) and on patients, and collected after screw placement had been finished.

### Statistical methods

SPSS Statistics 20 (IBM, Armonk, NY) was used for data analysis. Statistical analysis was performed using chi-squared test for comparison of categorical parameters, t-test for comparison of continuous normally distributed parameters and Pearson´s correlation coefficient for calculation of correlations. A two sided p-value < 0.05 was considered to be statistically significant.

## Results

Compared to surgeon and other OR personnel, patients’ radiation exposure was higher for all measured body areas with both methods (Table 1).

**Table 1:** Radiation exposure values of several body regions of patient and OR personnel during spinal surgery in µSv, with p-values between conventional (C-arm) and navigation (O-arm) technique included.

Radiation Exposure (µSv)	Patient				p-value	Surgeon				p-value	Assisting Surgeon				p-value	Sterile Nurse				p-value	Radiology technician				p-value
	Conventional		Navigation			Convent.		Navigation			Convent.		Navigation.			Convent.		Navigation			Convent		Navigation		
	Mean	±SD	Mean	±SD		Mean	±SD	Mean	±SD		Mean	±SD	Mean	±SD		Mean	±SD	Mean	±SD		Mean	±SD	Mean	±SD	
Head	174	123	194	54	0.269	119	51	60	20	**<0.001**	131	163	69	27	0.114	95	42	64	30	0.015	82	28	61	30	**0.023**
Eye lens region	185	165	205	60	0.571	164	74	92	41	**<0.001**	129	93	84	36	**0.007**	92	37	68	20	**<0.001**	107	36	78	30	**0.005**
Thyroid region	587	1177	384	248	0.467	211	95	76	72	**<0.001**	123	40	86	34	**<0.001**	95	37	59	19	**<0.001**	106	29	83	26	**0.017**
Breast region	7742	14815	1150	1043	0.062	275	147	66	19	**<0.001**	95	34	65	21	**0.003**	97	55	59	23	**0.009**	81	34	58	18	**0.018**
Right hand						566	560	49	19	**<0.001**															
Left hand						321	222	44	23	**0.002**															
Gonad region	5505	9837	14249	11590	**0.019**																				
Surgery field	27757	31381	43254	19430	**0.022**																				

**Figure 1:**
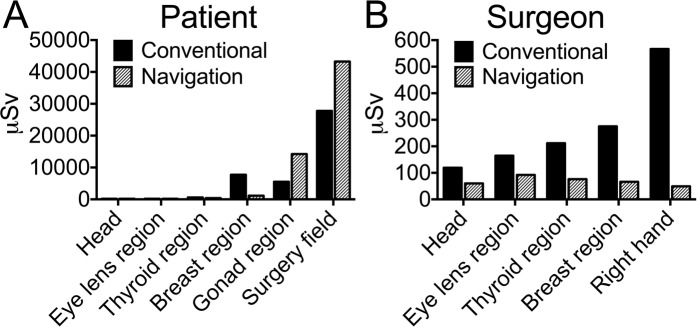
Radiation exposure of several body regions of patient (**A**) and surgeon (**B**) during spinal surgery in µSv, showing difference between conventional (C-arm) and navigation (O-arm) technique.

The highest radiation values on patients in this study were measured with the O-arm. The O-arm exposed the highest radiation dose on the surgery field, where the scans are performed and significantly higher dose compared to fluoroscopy was reached (navigation: 43.2 ± 19.4 mSv; fluoroscopy: 27.7 ± 31.3 mSv; p = 0.02). Followed by the gonad region, with high proximity to the scanned region (gonad region: navigation: 14.2 ± 11.5 mSv; fluoroscopy: 5.5 ± 9.8 mSv; p = 0.02). High values were also measured in the patient’s thoracic region, where exposure was significantly higher with fluoroscopy (navigation: 1.1 ± 1.0 mSv; fluoroscopy: 7.7 ± 14.8 mSv; p = 0.06). In comparison to these mentioned regions, the other measured patients’ body regions can be considered negligible (Table 1, Fig. 1A).

**Figure 2:**
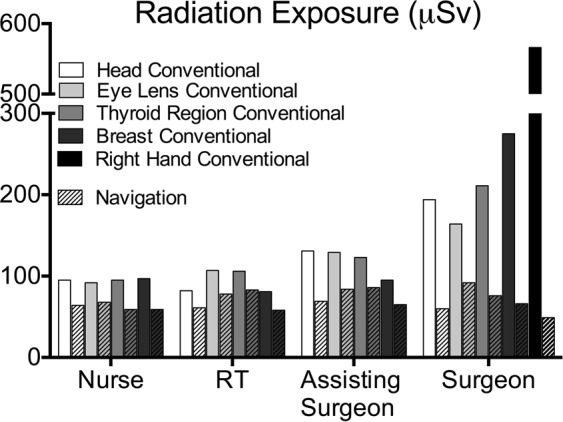
Radiation exposure of several body regions of sterile nurse, radiology technician (RT), assisting surgeon and surgeon during spinal surgery in µSv, showing difference between conventional (C-arm) and navigation (O-arm) technique.

Amongst the OR personnel, the operating surgeon was exposed to the highest radiation dose using fluoroscopy, whilst no difference to other personnel was measured during navigation (Table 1, Fig. 2).

Whilst navigation guided screw placement did not lead to divergent radiation exposure of the hands compared to other body regions, use of fluoroscopy led to highest surgeon’s exposure in this region (right hand: navigation: 49 ± 19 µSv; fluoroscopy 566 ± 560 µSv, p < 0.001; left hand: navigation: 44 ± 23 µSv; fluoroscopy 312 ± 222 µSv, p = 0.002) followed by thoracic region (navigation: 66 ± 19 µSv; fluoroscopy: 275 ± 147 µSv), thyroid and forehead (Table 1, Fig. 1B). Other OR personnel had comparable low radiation exposure doses on all measured body regions with both methods.

Focus on the eye’s lens region revealed that fluoroscopy led to highest exposure of eye’s lens region of the surgeon, whilst this value was significantly lower using navigation (fluoroscopy: 164 ± 74 µSv; navigation: 92 ± 41 µSv; p < 0.001). Patient eye’s lens exposure was higher than the surgeon’s in both methods (Fig. 2, Table 1).

There was a significant correlation between patient BMI and radiation exposure to the surgery field during fluoroscopy (*r* = 0.80, p = 0.01) which could not be found during navigation (*r* = 0.325, p = 0.18).

## Discussion

Since substantial health risk has been correlated to life time long radiation exposure of OR personnel, and highest radiation exposure levels have been found during spinal surgery, navigation based technologies have been established in order to reduce radiation exposure. To our knowledge, these data present the first real life, detailed comparison of radiation exposure on OR personnel and patients between clinical use of fluoroscopy and navigation.

We explored increased radiation dose exposed to patients using O-arm navigation, compared to decreased radiation dose exposure to the personnel, which is in concordance with earlier published surveys based on simulations^14^. Although radiation exposure to the patient during a single navigated spine surgery is up to 3 times higher compared to fluoroscopy guided cases, this, in most cases, singular event seems less significant compared to cumulative radiation exposure of a spine surgeon during a working life. Anyways, further appropriate dose reductions for medical exposures of the patients is still worthwhile and should be pursued whenever it seems possible.

A correlation between BMI and radiation exposure during fluoroscopy guided spinal surgery has been explored before, underlining the validity of our measurements^15^. Increased amount of soft tissue often leads to difficulties during screw placement in obese patients, demanding use of intraoperative fluoroscopy to a higher extent. These adjustments can be performed without additional radiation exposure during navigation, which could be an explanation why we could not detect this correlation in our navigation cases.

According to our data, radiation exposure of the surgeon’s body is significantly higher than for other OR personnel during fluoroscopy guided surgery. This is mainly determined by the surgeons’ proximity to the x-ray tube and varies inversely with the square of the distance.

Especially the surgeons’ dominant (in our case right) hand, which is mainly used to fix the screw position in the surgery field during fluoroscopy, received the highest radiation dose. We show in a clinical setting for the first time, that use of navigation technology significantly reduces the exposure on all body regions to the level of other personnel, since the OR is left during and reentered after the scan.

Our finding, that the operating surgeon’s eye lens region is exposed to significantly higher radiation doses compared to the assisting surgeon, can be explained by the surgeon’s position over the operative field during screw placement. This is further supported by the lack of radiation to the eye lens region when navigation is used. Although the patient’s eye lens exposure was even higher in both methods, it can be considered as less relevant, since mainly presenting a singular event.

Considering our sample as representative, a spinal surgeon could perform 10-fold (10.000 versus 883) surgeries using navigation before the maximum permissible annual radiation dose of the hand region (500 mSv per year) would be reached. Very similar values can be considered for the eye lens region.

We must highlight that TLDs were placed outside the lead aprons and collar during the study. Therefor we received unaltered radiation exposure of usually protected regions (thyroid, breast, gonad region) during surgery, which are directly comparable to usually not protected areas (head, eye’s lens region, and hands). Protective effects of a lead collar and lead apron were earlier demonstrated, reducing radiation dose by 96.9% on thyroid region, and 94.2% on breast and gonad region^16^. This implicates that radiation reduction and accurate protection must remain a main issue for a spinal surgeon, who is mainly exposed amongst OR personnel. In clinical routine, personal dosimeters are worn under the lead apron at our institution, evaluated monthly, and show dose values less than 0.2 mSv per month.

The use of navigation technology, also in standard cases, might be an effective tool concerning this topic. Our data also show that radiation exposure is mainly influenced by proximity to the source of radiation (Fig. 2, surgeon versus assisting surgeon) which should always be kept in mind during surgery.

### Study limitations

Anesthesia team members were not included in the study, although they are part of the OR personnel. They usually leave to the protected area outside the OR when radiation is performed and therefore were not considered to receive a relevant radiation dose.

The assisting surgeon usually tries to keep his hands out of the surgery field during fluoroscopic control and therefore radiation exposure was not measured in this study.

For measurements with TLDs as used in this study, a certain uncertainty, mainly due to the directionality of dosimeters and energy spectrum of the scattered radiation, must be expected. Nevertheless, this uncertainty did not alter our main findings, comparing radiation exposure of these imaging methods on OR personnel.

## Conclusion

Facing permanently increasing rates of radiation exposure due to imaging technology to personnel and patients, the caused risk should be part of the decision on which imaging method is chosen by the surgeon. The data presented in this study may improve risk evaluation, and raises several points:

1. Predominant use of navigation technology provides the opportunity of a significant work life dose reduction for the surgeon.
2. In any method used, distance and accurate protection must be the key elements to reduce the exposure rates below a dangerous value in the long run.
